# Whole-genome bisulfite sequencing data analysis learning module on Google Cloud Platform

**DOI:** 10.1093/bib/bbae236

**Published:** 2024-07-23

**Authors:** Yujia Qin, Angela Maggio, Dale Hawkins, Laura Beaudry, Allen Kim, Daniel Pan, Ting Gong, Yuanyuan Fu, Hua Yang, Youping Deng

**Affiliations:** Department of Quantitative Health Sciences, John A. Burns School of Medicine, University of Hawaii at Manoa, 651 Ilalo Street, Honolulu, HI 96813, United States; Health Data and AI, Deloitte Consulting LLP, 1919 N. Lynn Street, Arlington VA 22209, United States; Google Cloud, 1900 Reston Metro Plaza, Reston, VA 20190, United States; Google Cloud, 1900 Reston Metro Plaza, Reston, VA 20190, United States; Google Cloud, 1900 Reston Metro Plaza, Reston, VA 20190, United States; Health Data and AI, Deloitte Consulting LLP, 1919 N. Lynn Street, Arlington VA 22209, United States; Department of Quantitative Health Sciences, John A. Burns School of Medicine, University of Hawaii at Manoa, 651 Ilalo Street, Honolulu, HI 96813, United States; Department of Quantitative Health Sciences, John A. Burns School of Medicine, University of Hawaii at Manoa, 651 Ilalo Street, Honolulu, HI 96813, United States; Department of Quantitative Health Sciences, John A. Burns School of Medicine, University of Hawaii at Manoa, 651 Ilalo Street, Honolulu, HI 96813, United States; Department of Quantitative Health Sciences, John A. Burns School of Medicine, University of Hawaii at Manoa, 651 Ilalo Street, Honolulu, HI 96813, United States

**Keywords:** DNA methylation, WGBS, cloud computing, Google Cloud platform, epigenetics, bioinformatics education

## Abstract

This study describes the development of a resource module that is part of a learning platform named ‘NIGMS Sandbox for Cloud-based Learning’ https://github.com/NIGMS/NIGMS-Sandbox. The overall genesis of the Sandbox is described in the editorial NIGMS Sandbox at the beginning of this Supplement. This module is designed to facilitate interactive learning of whole-genome bisulfite sequencing (WGBS) data analysis utilizing cloud-based tools in Google Cloud Platform, such as Cloud Storage, Vertex AI notebooks and Google Batch. WGBS is a powerful technique that can provide comprehensive insights into DNA methylation patterns at single cytosine resolution, essential for understanding epigenetic regulation across the genome. The designed learning module first provides step-by-step tutorials that guide learners through two main stages of WGBS data analysis, preprocessing and the identification of differentially methylated regions. And then, it provides a streamlined workflow and demonstrates how to effectively use it for large datasets given the power of cloud infrastructure. The integration of these interconnected submodules progressively deepens the user’s understanding of the WGBS analysis process along with the use of cloud resources. Through this module, we can enhance the accessibility and adoption of cloud computing in epigenomic research, speeding up the advancements in the related field and beyond.

This manuscript describes the development of a resource module that is part of a learning platform named ``NIGMS Sandbox for Cloud-based Learning'' https://github.com/NIGMS/NIGMS-Sandbox. The overall genesis of the Sandbox is described in the editorial NIGMS Sandbox [1] at the beginning of this Supplement. This module delivers learning materials on the analysis of bulk and single-cell ATAC-seq data in an interactive format that uses appropriate cloud resources for data access and analyses.

## INTRODUCTION

DNA methylation is an epigenetic mechanism that involves the transfer of a methyl group onto the C5 position of the cytosine to form 5-methylcytosine [2]. This dynamic modification plays a crucial role in gene regulation and cellular differentiation during development, influencing tissue-specific gene expression patterns [3] and being linked to various diseases, including cancer and neurological disorders [4]. Advances in bisulfite-based technologies and next-generation sequencing (NGS) have enabled whole-genome bisulfite sequencing (WGBS), offering detailed information of epigenetic landscape and its implications for health and disease [5]. As an effective strategy, WGBS can identify individually methylated cytosines by treating genomic DNA with sodium bisulfite before sequencing [6]. Bisulfite treatment changes unmethylated cytosines to uracils, leaving methylated cytosines intact. This modification complicates WGBS data processing, as it requires mapping bisulfite-converted reads to a similarly converted reference genome for accurate alignment and methylation cytosine identification [6]. Many tools and workflows have been developed for the purpose of processing WGBS data [7], yet conducting the analysis is still not an easy task for non-bioinformaticians with limited experience in computational biology, and it usually requires high-performance computing resources with substantial computational power and storage.

Meanwhile, the biomedical field is looking for new methods and platforms to manage the vast volume of data generated in recent decades, exemplified by the WGBS datasets [8]. Processing such extensive datasets demands high-performance computing infrastructures and bioinformatic skills, posing challenges for many under-resourced institutions due to hardware costs and expertise limitations. As a viable solution, Cloud computing offers a secure, scalable environment, with essential resources and tools for data analysis and collaboration without substantial infrastructure investment. Although cloud services have been deployed for many tools and databases in biomedical science and related fields [9], they are still not widely accepted due to the learning curve associated with cloud computing. Therefore, there is a growing need for cloud-based educational resources, such as learning modules, that can equip researchers with the necessary skills and knowledge to leverage cloud computing effectively for biological data analysis.

In this article, we introduce a cloud-based learning module for WGBS data analysis designed for Google Cloud Platform (GCP), which comprises four submodules. Initially, it provides a detailed walkthrough using Bismark (v0.23.1) [10] to map bisulfite-treated sequencing reads to a reference genome and generate methylation levels for each cytosine position. Following this, metilene (v0.2.8) [11] was used for downstream analysis to identify differentially methylated regions (DMRs). Building on this foundation, the module incorporates nf-core/methylseq (v2.4.0) [12, 13] for a more streamlined WGBS data preprocessing workflow. Finally, to handle large-scale WGBS datasets, the last submodule leverages the power of GCP Google Batch, enabling researchers to optimize the analysis process by taking full advantage of GCP’s robust infrastructure. The use of real-world case studies demonstrates the practical application of cloud-based resources for managing large datasets, which highlights the cost-effective optimization strategies. This comprehensive module offers researchers the skills needed for cloud-based epigenetic data analysis and sets a foundation for them to move forward to more advanced topics in the related field.

## METHODS AND IMPLEMENTATION

The WGBS learning module, including its source code and data, is publicly available on the National Institute of General Medical Sciences (NIGMS) GitHub repository: https://github.com/NIGMS/DNA-Methylation-Sequencing-Analysis-with-WGBS. It can be downloaded directly using ‘*git clone*’ command to a GCP Vertex AI Workbench notebook or similar environment. The GCP Vertex AI offers a Jupyter Lab environment to simplify machine learning development. While this module may not include any machine learning components, we can still take advantage of the Vertex AI’s interactive environment and its fully managed, scalable infrastructures with customization capabilities. Detailed instructions for setting up and using the module are available in the README file provided with the module. These instructions cover setting up a GCP project, creating a billing account, enabling APIs, configuring a Nextflow service account and creating a Vertex AI notebook instance for downloading and executing the learning module.

The module includes an introductory document for DNA methylation data analysis and four interactive data processing submodules using Jupyter Notebook [14]. The Notebook environment supports interactive code execution and dynamic visualization of results, and it also provides space for clear explanations and the flexibility to explore alternative options. Quizzes and flashcards are also included in the module to aid knowledge retention and reinforce key concepts.

**Figure 1:**
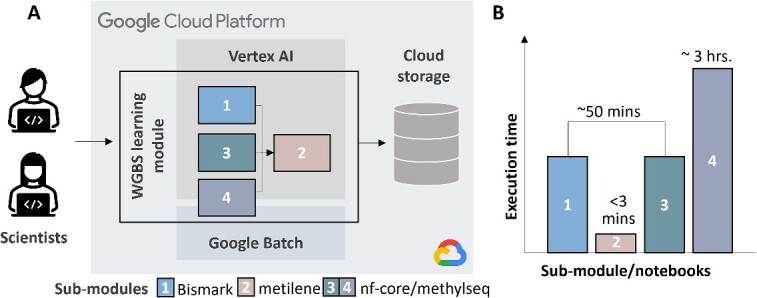
WGBS cloud-based learning module architecture. (A) The design architecture of the four interconnected submodules with different tasks and cloud environments. (B) The running times for each submodule to provide guidance of effective learning.

The four data-processing submodules (notebooks) are interlinked to form a comprehensive WGBS data analysis workflow, as illustrated in Figure 1A. Submodules 1, 3 and 4 focus on preprocessing raw WGBS data to determine genome-wide methylation levels, while submodule 2 utilizes these results to identify DMRs. In submodule 1, the Bismark tool processes data to derive methylation levels, feeding into submodule 2 (metilene module) for DMR identification through comparative analysis. Submodules 3 and 4 further refine the preprocessing workflow by utilizing the Nextflow nf-core/methylseq pipeline. This optimized one-command approach mirrors the functionality of Bismark, while incorporating additional quality control steps. While submodules 1 and 3 use a smaller, illustrative dataset stored in Google Cloud Storage for workflow demonstration, submodule 4 expands the analysis to a larger real-world dataset, accessible via SRA Toolkit. This escalation in data size highlights the utility of Google Batch’s in submodule 4, enabling scalable and more efficient processing of large datasets. The dataset for submodules 1 and 3 is stored in a GCP Cloud Storage Bucket (gs://nigms-sandbox/dna-methyl), which can be accessed using several methods, such as Google Cloud console and gcloud, gsutil commands.

The execution time for running the four submodules varies, as illustrated in Figure 1B. The preprocessing submodules 1 and 3 both process the same small exemplary WGBS dataset using the default machine type provided in Vertex AI Workbench (Python3 image on an n1-standard-4 machine with 4 vCPUs and 15 GB RAM), and can complete the jobs in approximately 50 minutes. Using the same machine, submodule 2, the metilene module, requires only 2–3 minutes to execute all steps to identify DMRs using the methylation profiles generated from submodule 1 as input. In contrast, submodule 4, the large-scale module, demands more time, approximately 3 hours, due to the downloading and processing of the larger dataset compared to the exemplary data used in the previous submodules. Notably, this large-scale module is executed using Google Batch, and attempting to run it on the default notebook machine type will fail because it demands more memory and storage than the default settings can accommodate.

All the submodules start by installing necessary tools and software, where Conda (https://docs.anaconda.com) and Mamba (https://github.com/mamba-org/mamba) are used to make the installation process easier. Conda is an open-source package and environment management system which can quickly install, run and update packages along with their dependencies. Mamba, implemented in C++, serves as a faster and more efficient alternative to Conda. Conda comes pre-installed in the Vertex AI notebooks and can be used directly. However, if it is not installed (such as in a self-managed virtual machine), installation instructions are provided in the submodules.

### Submodule 1: Bismark module: step-by-step pre-processing of WGBS data

**Figure 2:**
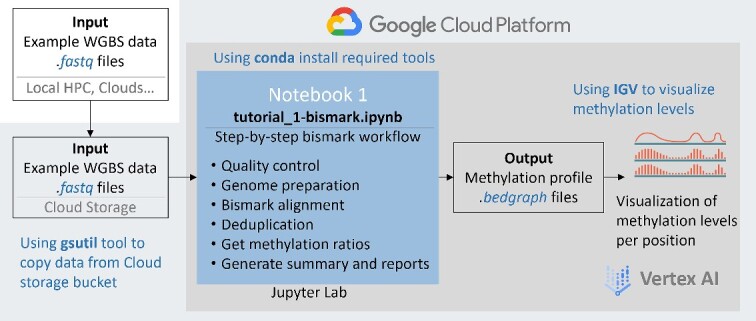
The design of the Bismark module, a step-by-step tutorial for WGBS data pre-processing. The input files can be uploaded directly or from Cloud Storage. The output results are DNA methylation levels at each cytosine position and can be viewed using IGV.

The Bismark module involves a step-by-step Bismark workflow using a small WGBS dataset stored in a Cloud Storage bucket (Figure 2). The Bismark workflow is widely used in the field of DNA methylation sequencing analysis not only for WGBS but also supports reduced-representation bisulfite sequencing and Post-Bisulfite Adapter Tagging (PBAT-Seq). The primary objective of this submodule is to provide users with a detailed guide to the Bismark workflow, including essential steps such as quality control, adapter removal, bisulfite conversion of the reference genome, bisulfite alignment, deduplication and methylation calling.

As shown in Figure 2, with necessary tools installed, the first step of this workflow is to download the example dataset and reference genome from Google Cloud Storage bucket using *gsutil* commands. If users would like to use their own datasets, they can upload the data to a storage bucket they create. To pre-process the data, Bismark workflow consists of several major steps. First, quality control is performed to ensure the reliability and accuracy of the data. FastQC (v0.11.9) [15] and MultiQC (v1.10.1) [16] are used for visualization of the data quality, followed by the removal of unnecessary adapters and low-quality sequences using Trim Galore! (v 0.6.7) [17]. To correctly map the qualified WGBS reads to a reference genome, the reference genome needs to be bisulfite converted as well. The most time-consuming step is when the reads are aligned to the converted reference genome by *bismark* command using the default aligner Bowtie2 [18]. Compared to the normal alignment SAM format, the Bismark alignment output format contains a tag of methylation call string, which is explained in detail in the submodule. After removing duplicate reads, methylation levels are finally called to for each genomic location with methylated or unmethylated cytosines. The methylation levels, also known as methylation ratios, are the output of this submodule, and for each cytosine position, the ratio is calculated as the percentage of methylated cytosines among all cytosines mapped to that specific position.

To summarize the data processing in this submodule, Bismark offers a graphical HTML report for each sample based on the outputs generated from all the major steps, such as alignment, de-duplication and methylation extraction. For visualization of the final DNA methylation patterns, the Python package ‘*igv-notebook’* is used to integrate the Integrative Genomics Viewer (IGV) [19] into Jupyter notebooks. IGV serves as a robust genome browser to explore genomic data, including DNA sequences, gene annotations, and epigenetic modifications. In this submodule, we introduce the basic usage of IGV, covering topics such as installation, loading data tracks and visualizing different data types, including the bisulfite alignment and methylation ratios obtained from the Bismark workflow.

### Submodule 2: downstream analysis for DMR identification using metilene

Following an initial pre-processing of a DNA methylation dataset, the next typical step is the identification of DMRs that consistently display distinct DNA methylation levels between different sample groups [20]. These regions (DMRs) vary in size, ranging from individual cytosine to entire gene loci, depending on the biological question of interest and the specific bioinformatic methods used for their detection. While some individual methylated sites may be associated with gene expression regulation and disease risk, the majority of reported DMRs in scientific literature typically fall within a size range of a few hundred to a few thousand bases, which corresponds to the typical dimensions of gene-regulatory regions [21].

The second submodule in our WGBS learning module focuses on identification of DMRs using metilene as the downstream analysis of WGBS data. Metilene is designed to identify and annotate DMRs, as well as differentially methylated CpG sites (DMCs) from methylation sequencing data. It considers intra-group variances and offers multiple modes to detect different types of DMRs, including *de novo* DMR detection, DMR detection within a known set of genomic features and DMC detection. The submodule introduces the first two modes focusing on regions instead of individual cytosine positions.

**Figure 3:**
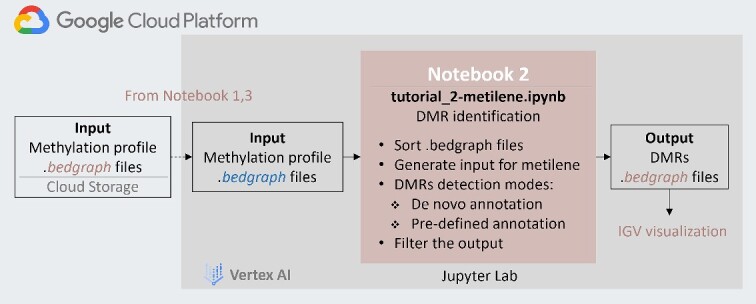
The workflow of the metilene module illustrates the downstream analysis to identify differentially methylated regions (DMRs). The module takes input files from the preprocessing modules (1 or 3). A dashed line indicates optional steps that can be skipped if bedgraph files are already present in the notebook environment. Two modes for detecting DMRs are introduced: de-novo annotation and predefined annotation.

In the metilene submodule, the initial step is transforming the methylation profiles obtained from the pre-processing module (Figure 3) to fit the format requirement of metilene. This transformation includes sorting the profiles based on chromosome and start position, followed by assigning sample groups to them to facilitate comparative analysis using a Perl script included in the metilene software. The first DMR detection mode introduced is the default mode of metilene, performing de-novo annotation of DMRs without relying on any prior genomic feature information, such as promoter or enhancer regions. This mode utilizes a rapid circular binary segmentation approach to pinpoint regions displaying maximum differences in the cumulative sum of mean methylation variations, thereby identifying potential DMRs [11]. The potential DMRs undergo statistical filtering and testing to confirm their significance, resulting in the generation of final validated DMRs. The output format of these DMRs is stored in the format of *bedgraph* files, where each DMR is annotated by its respective genomic position in the genome. In the second DMR detection mode, metilene identifies DMRs within a user-defined group of genomic features, such as genes, promoters or CpG islands. Unlike the default mode, this mode skips the circular binary segmentation algorithm and instead conducts statistical tests for each feature, reporting corresponding *P* values in the output. This pre-defined mode offers the flexibility to tailor DMR detection to their specific genomic features of interest, enabling a more targeted and in-depth analysis of epigenetic regulatory regions. Besides the usage of metilene, this submodule demonstrates how to obtain the genomic feature annotation files from other resources, such as the UCSC Genome Browser [22]. Following the selection of significant DMRs and various filtering options, the final DMRs from both modes can be visualized using IGV as well. Alongside this visualization, basic statistical plots characterizing the DMRs are generated by metilene, including distributions of DMR differences, DMR lengths in nucleotides, number of CpGs and DMR differences versus q-values.

### Submodule 3: streamlined pre-processing pipeline: Nf-core/methylseq

The first two submodules serve as introductions to the two fundamental aspects of WGBS data analysis, preprocessing and DMR detection. By engaging with these submodules, users should gain a thorough understanding of the intricacies of each processing step. Moving forward, the next objective is to provide a streamlined and efficient workflow, ensuring smoother WGBS data processing with minimal concern over individual steps. Therefore, the third submodule introduces the implementation of nf-core/methylseq pipeline [13], which allows users to prioritize reproducibility and scalability while focusing on their research findings.

**Figure 4:**
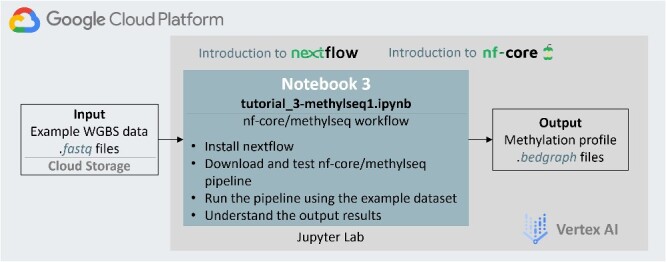
The design of the methylseq module, a streamlined WGBS pre-processing via nf-core/methylseq workflow. Fewer processing steps are needed in this submodule, due to the optimized one-command approach applied. Additional Nextflow and nf-core background information is included.

In this submodule (Figure 4), two essential concepts are introduced first: Nextflow [23] and nf-core [12]. Nextflow serves as a reactive workflow framework and programming domain-specific language (DSL), streamlining the creation of data-intensive computational pipelines. Nextflow enables the definition of complex program interactions and facilitates a high-level parallel computational environment based on the dataflow programming model. With this submodule, users can learn how to construct a straightforward ‘hello world’ Nextflow script and execute it. For the second concept, nf-core [12] is a collaborative community effort that compiles a curated collection of analysis pipelines leveraging Nextflow. Currently, nf-core has 100 available pipelines (as of Mar 2024), representing a full range of bioinformatics tools, such as analysis pipelines for RNA-seq, single-cell RNA-seq, ATAC-seq, ChIP-seq, etc. Therefore, mastering nf-core/methylseq in this submodule equips users with the skills to navigate other nf-core pipelines and use them confidently.

The nf-core/methylseq workflows can be downloaded and executed using a single Nextflow command, which specifies a profile (Docker, Singularity or Conda) to manage software dependencies. This command automatically retrieves the latest (or designated) workflow version and its dependencies, setting up an execution environment where the workflow runs and produces its output. To align the nf-core/methylseq pipeline’s execution with that of the Bismark submodule, several parameters need to be specified, including the input dataset location, reference genome, and an output directory. Upon completion, the output files from this streamlined workflow are compared with the results obtained from the Bismark module, which validates the effectiveness of the nf-core/methylseq pipeline.

### Submodule 4—advanced scaling: Nf-core/methylseq pipeline using Google batch

**Figure 5:**
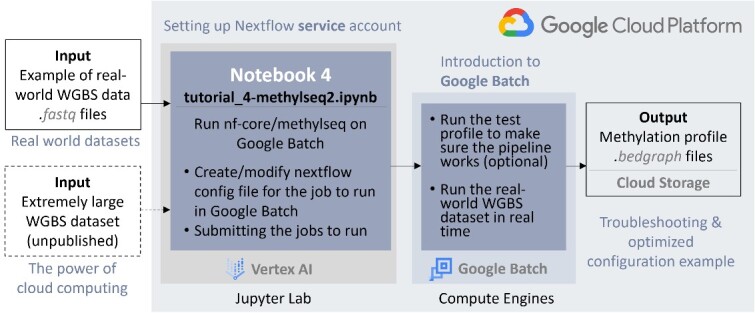
The design and key steps of the large-scale module, utilizing cloud resources for scalability. This submodule employs the nf-core/methylseq pipeline in conjunction with the Google Batch to process large-scale WGBS data

To address the resource-intensive nature of WGBS data analysis, the large-scale submodule exemplifies the potential of cloud computing for real-world WGBS data analysis. Analyzing WGBS data involves processing every cytosine in the genome to determine its methylation status, resulting in substantial data volumes and computational complexity. This submodule (Figure 5) provides an example using nf-core/methylseq via Google Batch to preprocess a WGBS dataset directly downloaded from the Sequence Read Archive (SRA). The utilization of cloud computing resources, such as high-performance machines optimized for specific purposes, enhances the efficiency of the analysis process, making it particularly advantageous for large-scale analyses.

Google Batch in GCP is a managed computing service that simplifies the execution of containerized workloads. It seamlessly integrates with Nextflow, allowing for easy deployment of pipelines like nf-core/methylseq, with process executions offloaded to Google Cloud’s powerful infrastructure. This is especially useful when comes to large-scale operations. To utilize Google Batch through Nextflow, users first need to create a Nextflow service account and grant permission to the Vertex AI notebook. The subsequent steps are similar to those in the methylseq submodule, with the addition of a new configuration file that specifies the executor, input/output directories, machine types, and the storage bucket to be used. These configurations can be set up individually for each step of the workflow, which allows for the use of different cloud computing resources based on specific tasks, enhancing cost-effectiveness.

As shown in Figure 5, the selected datasets are downloaded from SRA using SRA-tools and stored in the local notebook environment. The nf-core/methylseq pipeline is then executed on this dataset with the new configuration file, sending the job to the Google Batch for parallel execution using specified computation resources. Users can also define a local notebook directory using the parameter ‘*—tracedir*’ to save pipeline execution logs for tracing and runtime estimation. Once the process is complete, the output files are saved in the storage bucket defined in the configuration file.

As mentioned previously, to demonstrate the potential of cloud computing, we selected a moderately sized SRA dataset for this submodule. While this dataset allows the tutorial notebook to run without incurring excessive costs due to long runtime, it may not fully showcase the true advantages of GCP’s computational resources. To further test cloud-based analysis capabilities, we conducted thorough tests on a substantially larger dataset (not included in the submodule), providing valuable insights and experiences in optimizing configuration files and execution. By sharing our encountered problems and experiences in the submodule, we aim to assist users in making cost-effective decisions when using WGBS pipelines for their own large-scale data processing.

## RESULTS

The small example dataset used in submodules 1, 2 and 3 is from paper [24], subsampled by the snakePipes WGBS pipeline [25]. The original data with accession number GSE41923 is accessible through the NCBI GEO database. Four samples are selected from this dataset: two from mouse embryonic stem cells (ESCs) with two kinase inhibitors (2i) enabled derivations, and the other two from conventional ESCs maintained in serum. The sequences were further down-sampled to only contain the reads from the region: chr6:4000000–6,000,000 in the mouse genome.

**Figure 6:**
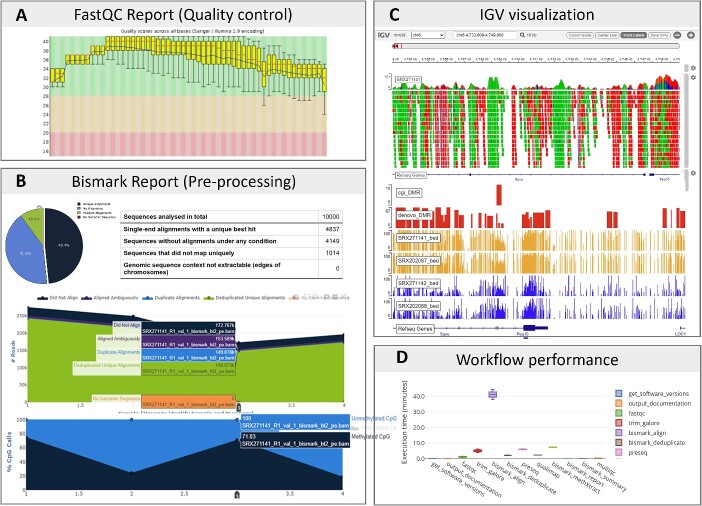
Examples of expected results from the WGBS learning module. (A) FastQC reports from the quality control step in Submodules 1 and 3, showing the quality score across different positions of the sequence reads. (B) Bismark report and summary examples for data processed in submodules 1 and 3, for all the steps in bismark workflow, including alignment, methylation calls for individual samples and a combined report summarizing the read number and average methylation level of all the samples. (C) Visualization using IGV to show bisulfite alignment (upper) from Submodule 1, where colors indicate different nucleotides, and methylation calling with DMRs (lower) from Submodule 2. (D) nf-core/methylseq workflow report from submodules 3 and 4, showing the execution time of each step in the pipeline.

Regarding the processing outcomes of this dataset using the WGBS learning module, every step generates output files, while certain steps yield reports intended for visualization purposes. Figure 6 shows the examples of the reports and visualizations that are generated from different steps across different submodules. Examining these outputs not only helps the users understand the data processing but also ensures everything is on track with no unexpected issues. For example, the quality control step provides insights into potential issues and biases in the WGBS data across samples, such as low quality, contamination and untrimmed adapters, through reports from FastQC (Figure 6A) and MultiQC. These reports serve as the foundation for trimming and filtering the sequences in the subsequent steps. The alignments can be visualized using IGV (Figure 6C, upper panel) to inspect the quality of the alignment and the distribution of methylated cytosines. The Bismark report and summary (Figure 6B) offer a thorough overview of the results obtained using Bismark, including mapping efficiency, methylation call rates and bisulfite alignment statistics. This report assists researchers in assessing the performance of the analysis steps and making informed decisions about further data processing or downstream analyses. Furthermore, the DMRs identified by metilene in submodule 2 can be visualized in a genome browser (Figure 6C, lower panel), helping validate the results and identify methylation patterns and trends. It also helps in data interpretation and hypothesis generation, such as uncovering potential regulatory regions in the genome. For the detected DMRs, metilene also creates basic statistic plots, such as distribution of DMR differences, DMR length in nucleotides and #CpGs. In the methylseq module, it produces an execution time report (Figure 6D) that offers a summary of the pipeline running parameters, resource utilization, and running time for each step. This report can help performance optimization, resource planning and troubleshooting.

From a biological perspective, the example WGBS dataset reveals a distinct methylation pattern, indicating that the samples from 2i ESCs exhibit global hypomethylation when compared to conventional ESCs maintained in serum (Figure 6C, lower panel). This is because the 2i treatment helps maintain ESCs in a more naive and undifferentiated state, which is associated with a lower overall DNA methylation level. However, the specific impact of 2i treatment on DNA methylation may vary depending on the genomic regions and genes involved. However, given the limitation of our small down-sampled dataset, the identified DMRs cannot fully capture all the changes in DNA methylation patterns induced by 2i treatment in ESCs.

The dataset used in the large-scale module originates from [26, 27], where the authors profiled the methylomes of human and chimpanzee sperm as a basis for comparison to methylation patterns of ESCs. It is important to note that this dataset serves primarily for testing the performance of the nf-core/methylseq pipeline in conjunction with Google Batch. While the large-scale module showcases the pipeline’s potential and efficiency, it does not include complete details of the preprocessing results and downstream analysis from this dataset.

In the large-scale module, we tested the actual benefits of cloud computing using an even larger dataset. This unpublished dataset consists of 12 samples, with 24 pair-end fastq files, with an average size of 325 M reads per file. We created an optimized config file for processing this large dataset with nf-core/methylseq and Google Batch, completing the analysis in under 27 hours. The optimization specifies different storage, memory, and number of CPUs to use for different processes in the nf-core/methylseq pipeline. This optimized configuration file is readily available with the learning module’s distribution. In contrast, running the same dataset on a local high-performance cluster, with maximum resources set to 256 GB memory and 32 CPUs, took over 5 days (134 hrs.). This substantial execution time difference highlights the remarkable efficiency and scalability of cloud-based computing for WGBS data analysis. This example can help researchers to make informed decisions, ensuring the most efficient utilization of resources and thereby elevating the cost-effectiveness and productivity of their analyses. These results provide compelling evidence for the advantages of cloud-based analysis, encouraging its broader adoption in the research community.

## DISCUSSION

The WGBS data analysis learning module effectively integrates WGBS tools and pipelines within the cloud environment (GCP), leveraging the cloud’s extensive computational resources and scalable environment to facilitate sophisticated data analysis and e-learning experiences. GCP’s robust infrastructure not only offers the necessary computational power but also supports a dynamic, interactive educational platform, allowing users to access, process and analyze large-scale genomic datasets efficiently. GCP’s commitment to data security is also critical in the context of handling sensitive DNA methylation data from clinical sources. GCP provides robust security features that comply with major certifications and regulations, ensuring that the data are processed and stored securely. These features include encryption of data in transit and at rest, identity and access management (IAM) to control user access to GCP resources and regular security audits for further reinforce the integrity and confidentiality of data. For this learning module, operational costs are low due to the relatively short runtime needed for tutorial datasets. However, comprehensive real-world analyses require advanced cost-management strategies. NIH CloudLab provides a cloud workspace for educational and training activities (limited time and budget), while NIH Cloud Initiatives support more extensive research with financial aid and resources. Crucially, for substantial data analyses, acquiring additional funding and grants is essential to cover the cost of cloud computing. Utilizing GCP’s cost-efficient features, such as sustained use discounts and customizable machine types, also helps in economizing. These combined approaches ensure that GCP remains an accessible, secure and efficient platform for genomic data analysis education and in-depth research.

As a comprehensive tutorial, this learning module begins with fundamental step-by-step data processing instructions. Users can not only learn how to analyze WGBS data and utilize cloud applications, but also acquire some programming and scripting skills, such as BASH and Nextflow. After users become familiar with the basic WGBS analysis steps, the module offers more advanced options, a more efficient and streamlined nf-core/methylseq pipeline and an optimized config file for running this pipeline on large scale datasets in the Cloud. The most exciting part is that, once users have fully mastered the usage of this nf-core pipeline, they will be able to explore and utilize other pipelines provided by the nf-core community, including diverse biological technologies beyond WGBS. This expertise will help researchers to broaden their analytical horizons across a wide range of biological studies. Last but not least, the module presents a powerful option for handling large-scale WGBS data using Google Batch, which requires a deeper understanding of the pipeline itself and the GCP Cloud resources.

While there are numerous tools available for downstream analysis of WGBS data, each with its strengths and applications, we have chosen to focus on metilene as one of the representative tools in this field. Metilene was selected for its fast and sensitive calling for DMRs, while alternatives like BSmooth [28], methylKit [29], methylSig [30], DSS [31], RADMeth [32], MOABS [33] and Biseq [34], etc., can all perform DMR identification based on different models. However, no single consistently outperforms others across all benchmark tests [35], which underscores the importance of exercising caution and diligence in data analysis and interpretation. There are also other tools and pipelines such as RnBeads [36] and msPIPE [37], which includes more visualization and gene functional analysis. It is important to note that the selection of metilene is not to undermine the significance of other tools in the field. Researchers are encouraged to explore other tools beyond the scope of this module to ensure a well-rounded perspective on WGBS data analysis, especially for different research needs. The learning module serves as a steppingstone, providing a solid foundation for users to try out other WGBS data analysis tools and uncover the complicated patterns of DNA methylation in their research.

During the development of the learning module, we have witnessed some major updates to critical tools used in it within a short span of one year. For example, the latest released version of nf-core/methylseq pipeline is 2.6.0 (as of March 2024), where we used version 1.6.1 during the development. And Google Batch is replacing Cloud Life Sciences API as a successor to manage batch services on Google’s infrastructure. These updates not only bring enhanced functionalities but also offer improved performance, scalability, and reliability. The continuous development and refinement of these tools demonstrates the dynamic nature of the technology field and the dedication of the bioinformatics community to stay at the forefront of cutting-edge research. It is essential to acknowledge that the landscape of WGBS data analysis and cloud computing will continue to evolve rapidly. While our learning module provides a foundation, researchers are encouraged to keep pace with the latest developments in tools and techniques. The learning modules are maintained and regularly updated under the auspices of NIH Cloud Lab, and many additional instructions are provided to address common queries and technical challenges. Users can contact the support team for direct assistance if they encounter any problems. Additionally, we are exploring the development of supplementary resources, including video tutorials and workshops to provide more support. These regular updates and maintenance will be crucial to ensure the smooth execution of workflows and to use cloud-based resources effectively.

This learning module, alongside other complementary modules, is made accessible to the public through NIH GitHub repository and maintained by the NIH Cloud Lab, offering a range of interactive and diverse training resources for students and researchers in the biomedical field. Besides WGBS data, this learning platform also offers the analysis of other types of data, such as RNA-seq, ATAC-Seq, metagenomics data, along with other biomedical research topics. This effort holds special significance for the NIH Institutional Development Award (IDeA) states, which often face unique challenges in terms of research infrastructure and funding opportunities. In a broader context, these modules demonstrate how collaborative efforts between the private sector, academic institutions, and federal agencies play a crucial role in driving scientific advancement. The collaborations of multidisciplinary teams from Google, Deloitte, NIH and researchers from IDeA states, have made this whole learning module development seamless and efficient. This convergence of expertise from industry, consulting, governmental sectors and academia exemplifies the power of diverse perspectives and resources in shaping elaborate educational frameworks.

## CONCLUSION

This comprehensive WGBS data analysis learning module, by integrating data analysis tools with cloud computing, provides researchers a powerful and efficient platform to explore the DNA methylation patterns through the analysis of WGBS data. Following a series of step-by-step submodules, users can acquire essential skills in WGBS data pre-processing, downstream analysis and using GCP cloud environment and resources. The utilization of nf-core/methylseq pipeline in conjunction with the Google Batch demonstrates the module’s potential in delivering scalability, cost-effectiveness and high-performance computing capabilities. Its development underscores valuable lessons, including the significance of integrating specialized tools with cloud computing, the importance of progressive learning for non-experts, careful tool selections and adaptability to rapid technological changes. We hope that this module will have an impact on the scientific research community in this rapidly evolving field.

**Key Points**

- This module introduces an in-depth WGBS data analysis module hosted on GCP, designed to equip researchers with essential skills in handling and analyzing WGBS data.
- It incorporates a series of interconnected workflows that guide learners through various stages of WGBS data analysis using cloud resources, from preprocessing to the identification of DMRs, with interactive and hands-on approaches to enhance learning.
- The module emphasizes the use of cloud computing and its effectiveness to handle large datasets, making advanced data analysis accessible to researchers without the need for extensive local computing resources.
- The learning module along with other NIGMS Sandbox modules provides a teaching framework of data sciences skills for students and researchers to utilize the power of cloud technology.

## Data Availability

The WGBS module is available at NIH GitHub repository: https://github.com/NIGMS/DNA-Methylation-Sequencing-Analysis-with-WGBS. The small example WBGS dataset and its reference genome used in submodules 1, 2 and 3 are stored in Google Cloud Storage Bucket (gs://nigms-sandbox/dna-methyl). The SRA dataset used in submodule 4 can be downloaded using their accession numbers: SRR306435 and SRR033942.

## Notes

**Dr. Yujia Qin**, a bioinformatician at the University of Hawaii JABSOM, earned her Ph.D. in Microbiology and a master’s degree in computer science from the University of Oklahoma, USA.
